# Development of a risk-adjusted in-hospital mortality prediction model for community-acquired pneumonia: a retrospective analysis using a Japanese administrative database

**DOI:** 10.1186/1471-2466-14-203

**Published:** 2014-12-16

**Authors:** Hironori Uematsu, Susumu Kunisawa, Noriko Sasaki, Hiroshi Ikai, Yuichi Imanaka

**Affiliations:** Department of Healthcare Economics and Quality Management, Graduate School of Medicine, Kyoto University, Yoshida Konoe-cho, Sakyo-ku, Kyoto City, Kyoto, 606-8501 Japan; Department of Biomedical Sciences, Ritsumeikan University, Norohigashi, Kusatsu City, Shiga, 525-0058 Japan

**Keywords:** Community-acquired pneumonia, Risk-adjusted mortality, Prognosis prediction model, Severity index, Scoring system, Administrative database

## Abstract

**Background:**

Community-acquired pneumonia (CAP) is a common cause of patient hospitalization and death, and its burden on the healthcare system is increasing in aging societies. Here, we develop and internally validate risk-adjustment models and scoring systems for predicting mortality in CAP patients to enable more precise measurements of hospital performance.

**Methods:**

Using a multicenter administrative claims database, we analyzed 35,297 patients hospitalized for CAP who had been discharged between April 1, 2012 and September 30, 2013 from 303 acute care hospitals in Japan. We developed hierarchical logistic regression models to analyze predictors of in-hospital mortality, and validated the models using the bootstrap method. Discrimination of the models was assessed using c-statistics. Additionally, we developed scoring systems based on predictors identified in the regression models.

**Results:**

The 30-day in-hospital mortality rate was 5.8%. Predictors of in-hospital mortality included advanced age, high blood urea nitrogen level or dehydration, orientation disturbance, respiratory failure, low blood pressure, high C-reactive protein levels or high degree of pneumonic infiltration, cancer, and use of mechanical ventilation or vasopressors. Our models showed high levels of discrimination for mortality prediction, with a c-statistic of 0.89 (95% confidence interval: 0.89-0.90) in the bootstrap-corrected model. The scoring system based on 8 selected variables also showed good discrimination, with a c-statistic of 0.87 (95% confidence interval: 0.86-0.88).

**Conclusions:**

Our mortality prediction models using administrative data showed good discriminatory power in CAP patients. These risk-adjustment models may support improvements in quality of care through accurate hospital evaluations and inter-hospital comparisons.

## Background

Community-acquired pneumonia (CAP) is one of the most common infection-associated diseases, and is a major cause of hospitalization and death. In particular, older persons are particularly susceptible to CAP and pneumonia-related complications [1]. Thus, improvements to the quality and outcomes of CAP treatment would be expected to have considerable benefits to the quality of life in older persons [2].

Inter-hospital comparisons of the quality of care can support the assessment and improvement of hospital management [3]. Due to the inherent variations in patient disease severity among hospitals, inter-hospital comparisons should involve the evaluation of risk-adjusted performances that can distinguish between disease severity effects from care effects [4]. As patient mortality is one of the most important outcomes of CAP care, the development of accurate risk-adjusted mortality prediction models would facilitate hospital performance evaluations and aid inter-hospital comparisons.

Several pneumonia mortality prediction models have been developed for use in clinical settings, and the primary measure of model discrimination is the c-statistic. The most reliable scoring systems currently for predicting mortality in CAP patients are CURB-65 [5] (which was modified from an earlier version developed by the British Thoracic Society) and the pneumonia severity index (PSI) [6]. However, CURB-65 does not take into account patient comorbidities [7], and PSI includes factors that are not routinely examined, such as arterial pH. In addition, it is possible that these models need further improvement: a meta-analysis has reported the c-statistic values for CURB-65 and PSI to be 0.80 and 0.81, respectively [8]. The A-DROP scoring system, which is a modified version of CURB-65 developed by the Japanese Respiratory Society, has a higher level of discrimination than both CURB-65 and PSI, with a reported c-statistic of 0.85 [9].

Administrative databases comprising patient billing records have potential applications in the development of useful risk-adjusted mortality prediction models. These databases provide a wide variety of variables for analysis, and the large quantity of real-world data accords a degree of external validity to results. Rothberg et al. reported that their risk-adjustment model for predicting mortality in pneumonia patients based on administrative data showed good discrimination (c-statistic: 0.85) [10]. Although administrative databases do not usually include detailed clinical information, risk-adjustment models such as CURB-65 or PSI do incorporate such information, and are generally considered to be the gold standard in pneumonia mortality prediction. However, recent modifications to a Japanese multicenter administrative claims database have included the incorporation of clinical data according to the A-DROP scoring system. This addition of clinical data to the administrative database may support the development of more accurate risk-adjustment models for comparing hospital performances.

As patient charts contain details on standard predictors of mortality (such as vital signs and clinical test results), the prediction of patient mortality using chart review analyses are thought to be more accurate than those based on administrative data. However, the collection of data from multiple institutions for chart review analysis is labor-intensive and costly, thereby limiting its applications in large-scale inter-hospital comparisons.

The purpose of our study was to develop and validate a more accurate and practical risk-adjustment model to predict 30-day in-hospital mortality in CAP patients using factors available from a Japanese administrative database.

## Methods

### Data source

Patient-level data were obtained from the Quality Indicator/Improvement Project (QIP)—a project that involves the periodic collection of administrative claims data from voluntary participant acute care hospitals in Japan. The collected data are used in the subsequent analysis of healthcare processes, patient outcomes, and disease management [11]. In 2014, there were 388 hospitals participating in the QIP. These hospitals varied in scale, region, and healthcare provider type.

All participant hospitals provide data to the QIP according to the Japanese Diagnosis Procedure Combination (DPC) system format, details of which have been described elsewhere [12]. Briefly, the DPC system is a case-mix classification system for reimbursements to acute care hospitals in Japan under the public medical insurance scheme.

DPC data contain discharge clinical summaries and administrative claims information. Clinical summary data include hospital identifiers, patient demographics, discharge statuses, major diagnoses, and comorbidities. Diseases are identified through International Classification of Diseases, 10th Revision (ICD-10) codes. Clinical information to determine pneumonia severity according to the A-DROP system is also included. The administrative information component includes the type, number, and date of clinical procedures performed.

### Study sample inclusion and exclusion criteria

**Figure 1:**
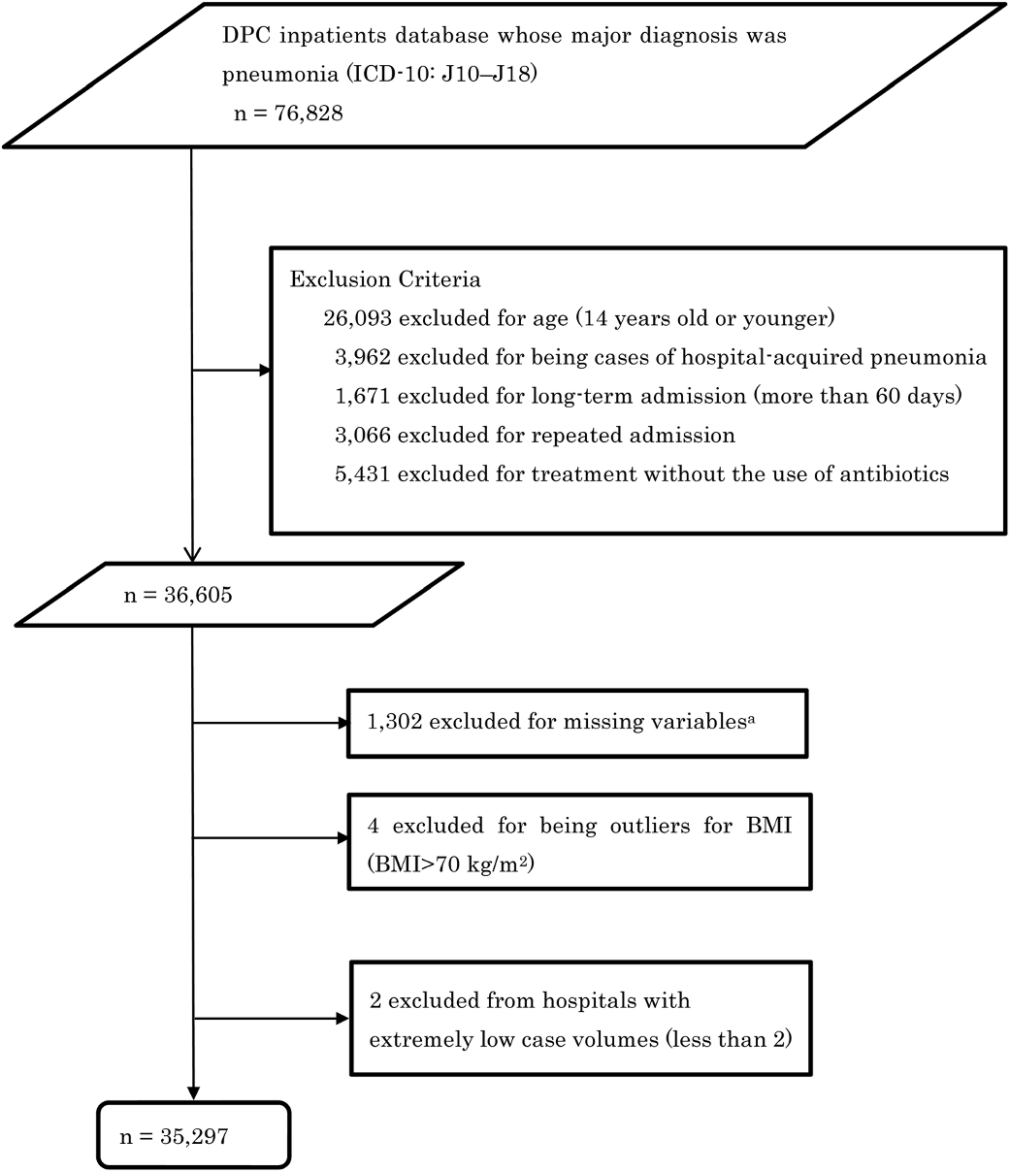
**Patient selection from the Diagnostic Procedure Combination (DPC) database.** ^a^missing variable included blood urea nitrogen or dehydration, respiratory state, orientation, blood pressure, C-reactive protein or extent of pneumonia infiltration, and immunodeficiency.

The patient selection process is presented in Figure 1. We selected inpatients who had been discharged (including mortality cases) from the study hospitals between April 1, 2012 and September 30, 2013, and whose major diagnosis for admission was pneumonia (ICD-10: J10–J18). Patients were excluded if they were aged 14 years or younger, had hospital-acquired pneumonia, repeated admission, long-term hospitalization (>60 days), or had not been administered an antibiotic within 2 days of admission. Patients with missing data for all study variables except for body mass index (BMI) were excluded from analysis. Patients were also excluded if they had been admitted to a hospital with a pneumonia case volume of only one patient during the study period.

### Statistical analysis

The primary endpoint was 30-day in-hospital mortality. Patients were divided into a 30-day in-hospital mortality group and a control (survival) group, and the baseline patient characteristics of the 2 groups were compared using Mann–Whitney U test or chi-squared test, as appropriate.

Using 30-day in-hospital mortality as the dependent variable, a hierarchical logistic regression model was developed with patients at the first level and hospitals at the second level. We employed a random intercept model with hospitals as random effects. We explored the following candidate independent variables as fixed effects: patient age, sex, BMI, vital signs, orientation disturbance (assessed to be present if a patient’s Japan Coma Scale [JCS] [13] score was 1 or more), extent of pneumonia infiltration, dehydration, comorbidities, ambulance use, and life support procedures such as mechanical ventilator use, vasopressor use, and hemodialysis. The attending physicians determined the extent of chest X-ray infiltration (if any) and recorded the result on this evaluation in the administrative database, detailing whether the infiltration covered more than two-thirds of the lung. Comorbidities included cancer (primary cancer and metastatic cancer), liver disease, renal disease, congestive heart failure, cerebrovascular disease, and immune deficiency. The Dartmouth-Manitoba version of the Charlson Comorbidity Index was used to identify comorbidities [14] (except immune deficiency) through corresponding ICD-10 codes. The cut-off points to stratify continuous variables such as age, BMI and C-reactive protein (CRP) were determined based on values reported in the existing literature [15, 16]. Discrimination of the logistic regression model was evaluated using the c-statistic [17], and internal validation was assessed using the bootstrap method. We performed 1,000 bootstrap resamples to calculate the odds ratios (ORs) of the predictors and c-statistics of the models [18].

Strong predictors of mortality were selected from the hierarchical logistic regression model for use in the development of more refined models to predict 30-day in-hospital mortality; the predictors were chosen to allow accurate predictions of mortality with fewer independent variables. To compare our newly developed models with an existing validated model, we also developed and analyzed a mortality prediction model based on the A-DROP scoring system.

Finally, we created scoring systems in which each independent variable within a model was allocated a single point, and we calculated the regression coefficients and mortality rates in each cumulative score. We also compared c-statistics between our scoring systems and the A-DROP system using chi-squared tests. All statistical analyses were performed using SPSS software, version 20 (SPSS Inc., Chicago, IL, USA) and STATA 12 statistical software (STATA Corp, College Station, TX, USA).

### Ethical standard

The collection and analysis of DPC data from the QIP hospitals were approved (Approval number: E-05) by the Ethics Committee of Kyoto University Graduate School of Medicine, and informed consent was waived. This study complied with the Ethical Guidelines for Epidemiological Research stipulated by the Japanese national government, which include guidelines on protecting patient anonymity, and all the necessary conditions were satisfied for informed consent to be waived.

## Results

### Patient characteristics

We analyzed 35,297 patients with CAP from 303 hospitals. Table 1 presents the patient characteristics of the study sample. The mean patient age was 78 years, and 20,667 (58.6%) patients were male. Hypoxemia (SpO_2_ < 90%) was observed in 33.3% of the patients. Approximately one-fourth of the patients had been hospitalized through emergency admission via ambulance. The overall and 30-day in-hospital mortality rates were 6.9% and 5.8%, respectively, which were within the range reported in recent pneumonia registries [19].

**Table 1:** **Demographic characteristics of 35,297 pneumonia patients**

	Overall		30-day in-hospital survival cases		30-day in-hospital mortality cases		
Number of hospitals	N = 303						
Patient characteristics	n = 35,297		n = 33,256		n = 2,041		***P***
Median age, year (IQR)	78	(68–86)	78	(67–85)	85	(79–90)	< 0.001^a^
Male, no. (%)	20,667	(58.6)	19,359	(58.2)	1308	(64.1)	< 0.001^b^
Median BMI, kg/m^2^ (IQR)	20.7	(18.2-23.5)	20.8	(18.3-23.6)	19.1	(16.6-21.7)	< 0.001^a^
BUN ≥7.5 mmol/L or Dehydration, no. (%)	12,623	(35.8)	11,183	(33.6)	1,440	(70.6)	< 0.001^b^
SpO_2_ < 90%, no. (%)	11,753	(33.3)	10,423	(30.8)	1,510	(74.0)	< 0.001^b^
Orientation disturbance, no. (%)	6,116	(17.3)	5,189	(15.6)	927	(45.4)	< 0.001^b^
Systolic BP ≤90 mmHg, no. (%)	2,038	(5.8)	1,507	(4.5)	531	(26.0)	< 0.001^b^
CRP 200 mg/L or extent of consolidation on chest X-ray ≥2/3 of one lung, no. (%)	6,333	(17.9)	5,423	(16.3)	910	(44.6)	< 0.001^b^
Comorbidities, no. (%)							
Cancer	4,154	(11.8)	3,800	(11.4)	354	(17.3)	< 0.001^b^
Liver disease	242	(0.7)	229	(0.7)	13	(0.6)	0.784^b^
Renal disease	1,558	(4.4)	1,407	(4.2)	151	(7.4)	< 0.001^b^
Congestive heart failure	5,435	(15.4)	4,877	(14.7)	558	(27.3)	< 0.001^b^
Cerebrovascular disease	3,002	(8.5)	2,819	(8.5)	183	(9.0)	0.442^b^
Immune deficiency	2,404	(6.8)	2,167	(6.5)	237	(11.6)	< 0.001^b^
Emergency admission via ambulance, no. (%)	9,418	(26.7)	8,320	(25.0)	1,098	(53.8)	< 0.001^b^
Life support procedure, no. (%)							
Mechanical ventilator^c^	795	(2.3)	514	(1.5)	281	(13.8)	< 0.001^b^
Vasopressor^c^	552	(1.6)	305	(0.9)	1247	(12.1)	< 0.001^b^
Hemodialysis^c^	396	(1.1)	361	(1.1)	35	(1.7)	0.003^b^

^a^ *P* values by Mann–Whitney U test. ^b^ *P* values by chi-squared test.

^c^Ventilator and vasopressor treatments were started within 2 days of admission. Hemodialysis was started within 3 days of admission.

Abbreviations: IQR, interquartile range; BMI, body mass index; BUN, blood urea nitrogen; SpO_2_, pulse oximetry saturation; BP, blood pressure; CRP, C-reactive protein.

### Mortality prediction model for pneumonia

Table 2 shows the hierarchical logistic regression results for 30-day in-hospital mortality after bootstrap resampling. All predictors except the following were significantly associated with increased ORs for mortality: BMI ≥ 25 kg/m^2^, liver disease, renal disease, and cerebrovascular disease. Ordinal variables such as age, respiratory status, orientation, and blood pressure showed a dose–response relationship with in-hospital mortality. The c-statistic of the preliminary model was 0.896 (95% confidence interval [CI]: 0890–0.903), while the c-statistic of the model with bootstrap correction was 0.894 (95% CI: 0888–0.900), indicating a high level of discrimination.

**Table 2:** **Multivariable predictors of 30-day in-hospital mortality**

Variables	Adjusted odds ratio (95% CI)	***P***
Male	1.29 (1.15-1.45)	< 0.001
BMI (reference, ≥18.5 and <25 kg/ m^2^)	reference	
Missing BMI data^a^	1.93 (1.66-2.24)	< 0.001
<18.5 kg/m^2^	1.55 (1.36-1.77)	< 0.001
≥25 kg/m^2^	0.79 (0.63-0.97)	0.028
Age (reference, 15–64 y)	reference	
65–74 y	2.08 (1.55-2.79)	< 0.001
75–84 y	3.26 (2.50-4.25)	< 0.001
≥85 y	5.25 (3.99-6.91)	< 0.001
BUN ≥7.5 mmol/L or Dehydration	1.88 (1.67-2.12)	< 0.001
SpO_2_ (reference >90%, room air)	reference	
FiO_2_ < 35%^b^ (without Ventilator)	2.40 (2.09-2.75)	< 0.001
FiO_2_ ≥ 35%^b^ (without Ventilator)	6.63 (5.61-7.82)	< 0.001
Ventilator^c^	7.55 (6.00-9.52)	< 0.001
Orientation (reference JCS 0)	reference	
JCS 1-3	1.65 (1.43-1.90)	< 0.001
JCS 10-30	2.86 (2.30-3.55)	< 0.001
JCS 100-300	4.21 (3.20-5.53)	< 0.001
Systolic BP (reference > 90 mmHg)	reference	
≤90 mmHg (without Vasopressor)	2.96 (2.51-3.49)	< 0.001
Vasopressor^c^	4.24 (3.27-5.48)	< 0.001
CRP 200 mg/L or extent of consolidation on chest X-ray ≥2/3 of one lung	2.27 (2.00-2.58)	< 0.001
Comorbidities		
Cancer	2.25 (1.93-2.63)	< 0.001
Liver disease	1.38 (0.76-2.51)	0.286
Kidney (reference free)	reference	
Renal disease (without Hemodialysis)	1.20 (0.94-1.55)	0.149
Hemodialysis^c^	1.55 (0.95-2.54)	0.080
Congestive heart failure	1.43 (1.26-1.61)	< 0.001
Cerebrovascular disease	0.84 (0.68-1.02)	0.081
Immune deficiency	1.45 (1.20-1.76)	< 0.001
Emergency admission via ambulance	1.50 (1.33-1.69)	< 0.001
c-statistic	0.894 (0.888-0.900)	< 0.001

*Abbreviations:* CI, confidence interval; BMI, body mass index; BUN, blood urea nitrogen; SpO_2_, pulse oximetry saturation; FiO_2_, fraction of inspired oxygen; JCS, Japan Coma Scale; BP, blood pressure; CRP, C-reactive protein.

^a^BMI data missing: BMI data were missing in 4,698/35,297 pneumonia patients.

^b^FiO_2_ < 35% and ≥35%: fraction of inspired oxygen (<35% and ≥35%, respectively) required to maintain SpO_2_ > 90%.

^c^Ventilator and vasopressor treatments were started within 2 days of admission. Hemodialysis was started within 3 days of admission.

### Scoring systems for mortality prediction

Table 3 presents the results of 4 mortality prediction models with the adjusted ORs of 30-day in-hospital mortality. The models were developed using strong predictors of mortality identified from the results of the initial model presented in Table 2. These 4 models included an A-DROP model and 3 newly developed models (designated Models 0, 1, and 2). The A-DROP model was developed using variables of the existing A-DROP scoring system. In contrast to the A-DROP model, Model 0 and Model 1 excluded sex as a variable, and the cut-off ages were changed to 65 years and 80 years, respectively. Model 2 utilized the same predictors as Model 1, with the following 3 additional binary variables: CRP ≥ 200 mg/L or extent of consolidation on chest X-ray ≥ 2/3, use of mechanical ventilator/vasopressors, and presence of cancer. There were only low correlations observed between each of the variables in Table 3 (All Pearson’s coefficients were less than 0.22, P < 0.01). The c-statistics of Model 1 and Model 2 were 0.854 (95% CI: 0846–0.862) and 0.874 (95% CI: 0867–0.882), respectively.

**Table 3:** **Multivariable predictors of 30-day in-hospital mortality in each scoring system**

	Adjusted odds ratio (95% CI)			
	A-DROP ^a^model	Model 0	Model 1	Model 2
Number of predictors	5	5	5	8
Age ≥65 y		3.35 (2.15-2.97)		
Age ≥70 y (male) or Age ≥ 75 y (female)	2.52 (2.15-2.97)			
Age ≥80 y			2.00 (1.79-2.23)	2.43 (2.17-2.73)
BUN ≥7.5 mmol/L or Dehydration	2.59 (2.32-2.88)	2.61 (2.32-2.88)	2.59 (2.32-2.88)	2.15 (1.93-2.41)
SpO_2_ ≤ 90%	4.29 (3.84-4.79)	4.28 (3.83-4.79)	4.36 (3.90-4.87)	3.47 (3.09-3.89)
Orientation disturbance	2.70 (2.42-3.00)	2.77 (2.49-3.08)	2.62 (2.35-2.91)	2.46 (2.20-2.75)
Systolic BP ≤90 mmHg	4.12 (3.62-4.69)	4.08 (3.58-4.64)	4.18 (3.67-4.76)	3.23 (2.82-3.70)
CRP 200 mg/L or extent of consolidation on chest X-ray ≥2/3 of one lung				2.41 (2.16-2.69)
Mechanical ventilator or Vasopressor				4.18 (3.56-4.92)
Cancer				2.07 (1.81-2.38)
c-statistic	0.853 (0.845-0.861)	0.852 (0.844-0.860)	0.854 (0.846-0.862)	0.874 (0.867‒0.882)

*Abbreviations:* CI, confidence interval; BUN, blood urea nitrogen; SpO_2_, pulse oximetry saturation; BP, blood pressure; CRP, C-reactive protein.

^a^A-DROP: A, Age ≥70 y (male), ≥75 y (female); D, BUN ≥7.5 mmol/L; R, SPO_2_ < 90%; O, Orientation disturbance; P, Systolic BP <90 mmHg.

Table 4 presents the regression coefficients and 30-day in-hospital mortality rates for each score category of the A-DROP model, Model 1, and Model 2. In each model, the mortality rates increased together with increasing scores. The c-statistics for the scoring systems of the A-DROP model, Model 1, and Model 2 were 0.851 (95% CI: 0844–0.859), 0.850 (95% CI: 0842–0.858), and 0.871 (95% CI: 0864–0.879), respectively. Model 2 had a significantly higher c-statistic than the A-DROP model (P < 0.001), while there was no statistical difference between the A-DROP model and Model 1 (P = 0.464).

**Table 4:** **Adjusted logarithmic odds ratios and 30-day mortality in each score**

	A-DROP ^a^model			Model 1			Model 2		
	LOR (95% CI)	Death ^b^/Total	(%)	LOR (95% CI)	Death ^b^/Total	(%)	LOR (95% CI)	Death ^b^/Total	(%)
Score 0	reference	17/6,877	(0.2)	reference	44/10,059	(0.4)	reference	21/7,831	(0.3)
Score 1	1.80 (1.25-2.34)	156/10,543	(1.5)	1.62 (1.30-1.94)	220/10,148	(2.1)	1.59 (1.10-2.08)	125/9,687	(1.3)
Score 2	2.96 (2.43-3.49)	434/9,279	(4.5)	2.65 (2.35-2.96)	466/8,299	(5.6)	2.68 (2.22-3.14)	306/8,360	(3.7)
Score 3	4.13 (3.61-4.66)	707/4,967	(12.5)	3.75 (3.45-4.06)	677/4,945	(14.6)	3.60 (3.14-4.05)	460/5,477	(8.4)
Score 4	5.14 (4.61-5.68)	522/1,445	(26.5)	4.67 (4.36-4.98)	473/1,658	(28.5)	4.63 (4.17-5.09)	502/2,558	(19.6)
Score 5	6.59 (6.01-7.16)	205/350	(58.6)	6.12 (5.74-6.50)	161/268	(60.1)	5.56 (5.09-6.03)	393/1,054	(37.3)
Score ≥6							6.82 (6.31-7.32)	234/360	(65.0)
c-statistic	0.851 (0.844-0.859)			0.850 (0.842-0.858)			0.871 (0.864-0.879)		

*Abbreviations:* LOR, logarithmic odds ratio; CI, confidence interval.

^a^A-DROP: A, Age ≥ 70 (male), ≥ 75 (female); D, BUN ≥ 7.5 mmol/L; R, SPO_2_ ≤ 90%; O, Orientation disturbance; P, Systolic BP ≤ 90 mmHg.

^b^Death, 30-day in-hospital mortality.

## Discussion

In this study, we developed and internally validated risk-adjustment models and scoring systems for predicting in-hospital mortality in CAP patients using Japanese DPC data. In an analysis of 35,397 patients from 303 hospitals, our hierarchical logistic regression model demonstrated strong predictive power for CAP mortality with a c-statistic of 0.894 after bootstrap correction. This predictive power was comparable with existing mortality prediction models, regardless of whether they were based on chart reviews or administrative data [20]. In our model, we included unique variables not used in existing models, such as BMI, life support procedures, CRP, size of infiltration in chest X–ray, and ambulance use.

BMI below 18.5 kg/m^2^ was significantly associated with increased ORs for mortality relative to the BMI range of 18.5–24.9 kg/m^2^; in contrast, BMI of 25 kg/m^2^ or more was significantly associated with decreased mortality. This finding was consistent with the results of recent studies [15, 21]. However, a substantial number of cases in our sample had missing BMI data (n = 4,698), and these cases were found to be more strongly associated with increased mortality than any other BMI range. The reason for the missing data and its impact on mortality prediction should be addressed in the future.

The use of mechanical ventilation or vasopressors was strongly associated with increased mortality, although the proportion of patients in our sample who had undergone these procedures was relatively small. Ewig et al. [22] reported that the modified American Thoracic Society rule, which includes the requirement for mechanical ventilation or septic shock as a parameter, had excellent predictive power for pneumonia severity. In the practical clinical setting, however, life support procedures are not always conducted, especially in very elderly or terminal patients in consideration for their quality of life. If we were to analyze the requirement for mechanical ventilation or vasopressors in place of the use of these treatments as a variable for mortality prediction, the predictive power of the former may be higher than that of the latter due to a higher proportion of patients who qualify for these procedures but do not actually receive them. Therefore, the requirement for mechanical ventilation or vasopressors may be preferable as a variable than the actual use of these procedures.

Our use of a combination variable that integrated the parameters of CRP ≥ 200 mg/l and chest X-ray infiltrations covering at least two-thirds of one lung is unique among the international standard scoring systems. The individual associations of increased mortality with increased CRP level alone or infiltration on chest X-ray alone have previously been reported [16, 23, 24]. In our study, the combination variable showed a similar incidence and OR for mortality as orientation disturbance.

We believe that the severity scoring systems presented here can be used by healthcare organizations to evaluate healthcare and offer better quality of care. With an emphasis on practical usability, we sought to develop a scoring system with a high level of discrimination that utilizes a small number of variables. To this end, we developed and validated 2 scoring systems that are modified versions of the A-DROP scoring system.

The discriminatory power of the Model 1 scoring system was not statistically different from that of the A-DROP system, even though the former did not include patient sex as a variable. We posit that the lack of difference between the discriminatory powers of the 2 systems was due to a weak association between sex and mortality, as well as the superior predictive power of using a cut-off age of 80 years. The median age of our study population was 78 years, and a cut-off age of 80 years resulted in a more equal distribution of the sample. CURB-65, which uses a cut-off age of 65 years, was established in 2000 using a study population with a mean age of 64 years [5]. This underlines the importance of considering the appropriate cut-off age for the scoring system based on the age composition of the target population.

The Model 2 scoring system had better discrimination than the A-DROP system. The use of mechanical ventilation or vasopressors remained strongly associated with mortality even after adjusting for hypoxemia (SPO_2_ < 90%) and hypotension (Systolic BP < 90 mmHg). Therefore, the use of mechanical ventilation or vasopressors was given a weight of 1 point in our severity score, independent of respiratory failure or hypotension. In addition, Model 2 showed that cancer was a strong predictor for mortality among the comorbidities. Fine et al. have also acknowledged the strong association between cancer and mortality by giving the presence of cancer the highest score of all comorbidities in the PSI [25].

This study showed that our models were able to accurately predict mortality in CAP patients using only administrative data from within 2 days of hospital admission. Our risk-adjusted models may have applications in conducting more precise hospital performance evaluations and inter-hospital comparisons. Additionally, we hope these models may also have applications in developing more appropriate payment systems in the future that are able to take the different levels of pneumonia severity into account.

### Limitations

There are several limitations to this study. First, our samples may not be representative of all CAP cases because we did not include patients whose major diagnosis was either sepsis or respiratory failure with a secondary diagnosis of pneumonia. The inclusion of these patients may raise the overall or 30-day in-hospital mortality rates above those observed in our sample [26]. Furthermore, we did not exclude patients with diagnoses of interstitial pneumonia or exacerbation of chronic obstructive pulmonary disease imitating CAP. The difference in sampling range for pneumonia patients could therefore introduce misclassification bias to the study and potentially confound our results.

Second, the models developed in this study were only internally validated, and we did not conduct external validation using other data sets. However, we did use a large dataset of 303 hospitals with a variety of characteristics, which may improve the generalizability of the results. Furthermore, it is possible that a split-group validation may be more appropriate than the bootstrap method used here because many of the bootstrap samples were thought to have a degree of overlap due to the large size of the original cohort. More studies should be conduct to further validate our models before they can be used in practical settings.

Third, the database used in this study still lacked certain clinical variables that may improve our ability to predict mortality. Arterial pH, the level of serum sodium, serum albumin, and the presence of plural effusions have been reported to be strong predictors of mortality [25, 27], but were unavailable in our data.

Fourth, there are only 4 coding slots for comorbidities in the Japanese DPC database. Therefore, the incidence of comorbidities (such as cancer) in pneumonia cases identified in this study may be lower than actual incidences. Improving the coding system to allow more comorbidities to be recorded may enhance the quality of research based on these data.

Fifth, our study did not include a direct comparison of our models with the CURB-65 scoring system, which may be the most common method used to predict mortality in pneumonia; we were unable to calculate CURB-65 scores as our database did not include respiratory rate and blood urea nitrogen values as continuous variables, which are required parameters. However, apart from these two variables, our Model 0 was very similar to the CURB-65 scoring system.

Sixth, mortality in elderly patients or patients with terminal cancer can be affected by the degree of aggressive treatments that they receive. Predictors in our models may be confounded by this factor, as we could not acquire information regarding the degree of aggressive treatments received at the patient level in this study.

Finally, our data were obtained from acute care hospitals that voluntarily participate in the QIP. Therefore, there may be a degree of sampling bias that weakens the generalizability of our findings to chronic care facilities.

## Conclusions

In this study of 35,297 patients with CAP in Japan, we developed and internally validated risk-adjustment models and scoring systems for predicting in-hospital mortality using Japanese administrative data complemented with clinical data concerning pneumonia severity. Our models and scoring systems had superior discriminatory power over existing models, and may improve risk adjustments for inter-hospital comparisons and more accurate prediction of mortality in CAP patients.

## Abbreviations

CAP: Community-acquired pneumonia
PSI: Pneumonia severity index
DPC: Diagnosis procedure combination
QIP: Quality indicator/improvement Project
ICD-10: International Classification of Diseases 10th Revision
BMI: Body mass index
JCS: Japan coma scale
CRP: C-Reactive protein
ORs: Odds ratios.
